# Deletion of Indian hedgehog gene causes dominant semi-lethal Creeper trait in chicken

**DOI:** 10.1038/srep30172

**Published:** 2016-07-21

**Authors:** Sihua Jin, Feng Zhu, Yanyun Wang, Guoqiang Yi, Junying Li, Ling Lian, Jiangxia Zheng, Guiyun Xu, Rengang Jiao, Yu Gong, Zhuocheng Hou, Ning Yang

**Affiliations:** 1 National Engineering Laboratory for Animal Breeding and MOA Key Laboratory of Animal Genetics and Breeding, China Agricultural University, Beijing 100193, China; 2 Rural Energy Management Station of Guizhou Province, Guiyang, 550001, China; 3 Livestock Genetic Resources Management Station of Guizhou Province, Guiyang, 550001, China

## Abstract

The Creeper trait, a classical monogenic phenotype of chicken, is controlled by a dominant semi-lethal gene. This trait has been widely cited in the genetics and molecular biology textbooks for illustrating autosomal dominant semi-lethal inheritance over decades. However, the genetic basis of the Creeper trait remains unknown. Here we have utilized ultra-deep sequencing and extensive analysis for targeting causative mutation controlling the Creeper trait. Our results indicated that the deletion of Indian hedgehog (*IHH*) gene was only found in the whole-genome sequencing data of lethal embryos and Creeper chickens. Large scale segregation analysis demonstrated that the deletion of *IHH* was fully linked with early embryonic death and the Creeper trait. Expression analysis showed a much lower expression of *IHH* in Creeper than wild-type chickens. We therefore suggest the deletion of *IHH* to be the causative mutation for the Creeper trait in chicken. Our findings unravel the genetic basis of the longstanding Creeper phenotype mystery in chicken as the same gene also underlies bone dysplasia in human and mouse, and thus highlight the significance of *IHH* in animal development and human haploinsufficiency disorders.

One critical issue in biology is to understand the mechanism underlying phenotype formation. Monogenic traits make excellent models for linking phenotypes and genotypes. Chicken has been used as an important model organism for developmental biology, immunology and microbiology, leading to several fundamental discoveries in biology^1^. Many lethal mutations have been reported in chicken, but few of them have been cloned.

The semi-lethal Creeper trait, first described in 1925, was mainly characterized by a pronounced shortness of the extremities (Achondroplasia, OMIA000006-9031)^2^,^3^. It has been demonstrated that homozygous Creeper embryos generally die on the fourth day of embryonic development, but a few can survive to late stages^4^. Early histological studies indicated that lethal embryos have shown abnormalities in the vascular system, nervous system, limbs, and guts^5^,^6^. Genetic studies suggested that the Creeper trait was determined by a single autosomal dominant semi-lethal gene (*Cp*) in chicken. The *Cp/Cp* homozygotes are lethal during early embryonic development while the heterozygotes (Creeper) are viable with pronounced chondrodystrophy characteristic^6^,^7^. The linkage of genes for Creeper and single-comb was the first reported autosomal linkage case in fowls and farm animals^3^,^8^,^9^. The *Cp* gene was assigned to *Group A* in the first chicken linkage map^10^. It is closely linked to the Rose-comb gene (*R*)^9^,^11^,^12^,^13^ which is rearranged from the *MNR2* gene on chicken chromosome 7, leading to ectopic expression^14^. Due to its dominant semi-lethal characteristic and historic significance, the *Cp* gene has been widely cited in genetics and molecular biology textbooks^15^,^16^. However, the molecular basis of this phenotype is unknown.

In the present study, we have performed whole-genome sequencing to demonstrate that early embryonic death and the Creeper trait are caused by the deletion of Indian hedgehog (*IHH*), leading to a decreased expression of *IHH* during cartilage development, which is responsible for condensation, growth, and differentiation of cartilage. Our results dissect the molecular basis of the Creeper trait and early embryo death in chicken, and highlight the significance of *IHH* deletion as a dominant semi-lethal mutation in natural chicken populations. This study emphasizes the pivotal role of *IHH* in animal development and provides an ideal and valuable *in vitro* model for the study of *IHH* function and haploinsufficiency diseases.

## Results

### Xingyi bantam carries the *Cp* gene

**Figure 1:**
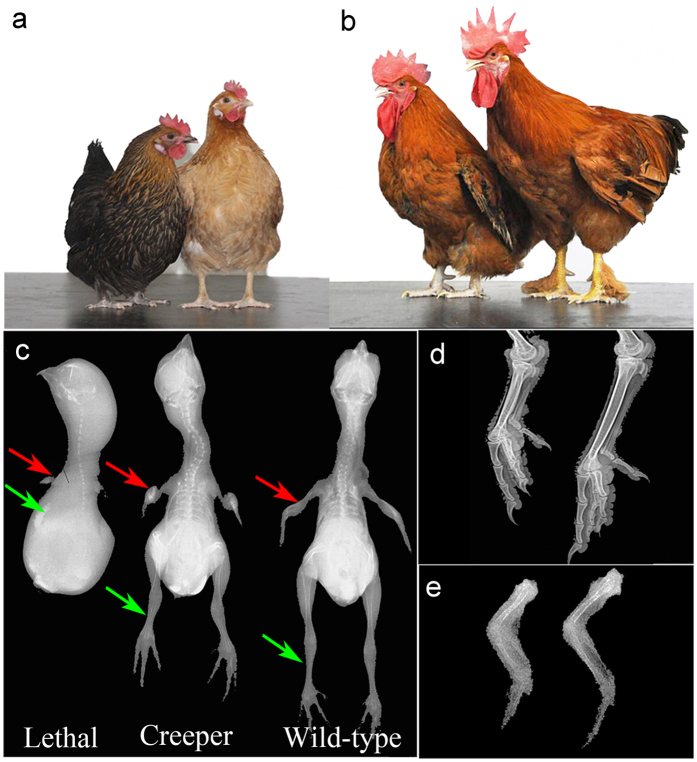
Phenotypes of Xingyi bantam chickens. (**a,b**) Female and male chickens. Left is the Creeper while right is the wild-type chickens. Shank length is shorter and body size is lower in the Creeper than in wild-type chickens. (**c**) Radiographic features of the lethal embryos (embryonic day 16) and day-old Creeper and wild-type chicks. Very small wings (red arrow) and legs (green arrow) were observed in the homozygous lethal embryos. (**d,e**) Comparison of shank (20 weeks) and wing (day-old chick) of the Creeper and wild-type chickens by digital radiography.

Chinese Xingyi bantam breed (Fig. 1a,b) is a valuable genetic resource characterized by the Creeper phenotype and good uniformity. In this study, we used Chinese Xingyi bantam as a model to target the causative mutation of the semi-lethal Creeper trait. During incubation of fertilized eggs, we observed 29.27% and 4.61% early embryonic mortality for the Creeper chickens and the control cross between Creeper and wild-type birds (Table 1). Embryonic mortality of Creeper chickens *inter se* was around 25% higher than that of the cross between Creeper and wild-type chickens. Ratio of Creeper and wild-type chickens followed 2:1 for Creeper chickens *inter se* (χ^2^ = 0.0229, *p* = 0.8796) while 1:1 for the crosses between Creeper and wild-type chickens (χ^2^ = 0.0292, *p* = 0.8643). In addition, three batch incubation experiments were performed to validate the segregation of Creeper chickens *inter se* and of the crosses between Creeper and wild-type chickens. The results were consistent with our first incubation experiment, as summarized in Supplementary Table S1. These results suggested that Creeper chickens were heterozygotes and the Creeper trait was controlled by a single dominant gene. The wing, shank and body size of the lethal embryos and Creeper chickens were significantly shorter/smaller than those of wild-type chickens (Fig. 1c–e and Supplementary Fig. S1), while body weight of the Creeper chickens was also lower than that of wild-type chickens from postnatal to adult stages (Supplementary Fig. S1).

The observed phenotypes of Xingyi bantam and segregation analysis results in the present study were consistent with previous experiments^7^,^17^. These findings further confirmed that Xingyi bantam breed carries the *Cp* gene.

### Deletion of *IHH* only exists in the Creeper chickens

**Figure 2:**
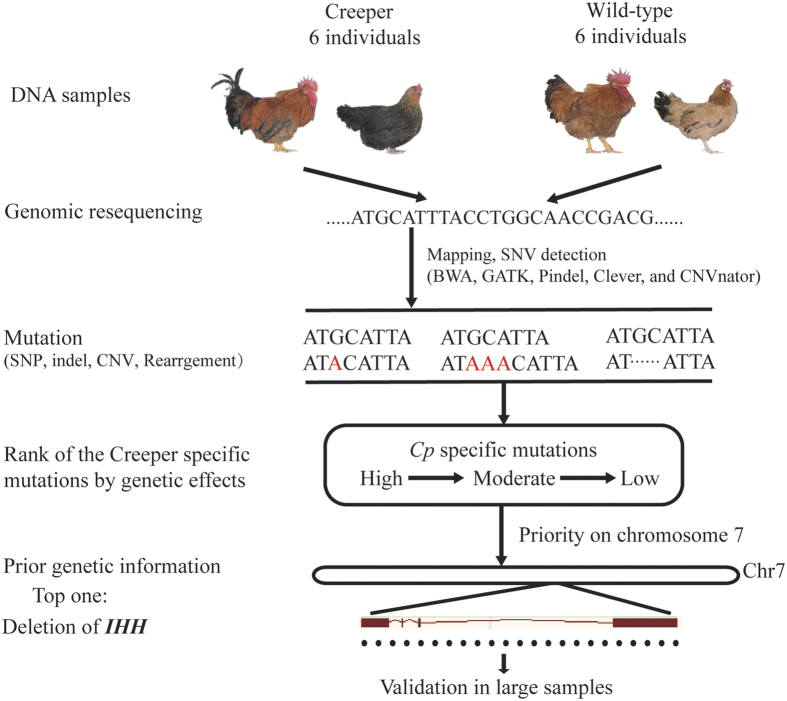
Pipelines for whole genome sequencing data analysis. Six pairs of full-sibs were obtained for whole-genome sequencing. Several current known major mutations were detected using various algorithms/software. Once the mutations were obtained, the mutations were assessed and ranked from high to low by their potential genetic effects.

In order to decipher the genetic basis of the semi-lethal Creeper trait in chicken, we constructed a segregation population from the pedigreed Creeper chicken mating (*Cp/*+ × *Cp/*+) and performed whole-genome sequencing of 6 pairs of full-sibs (6 Creeper chickens, *Cp/*+; 6 wild-type chickens, +/+) (Fig. 2). Average sequence coverage of 12-16X for all 12 individuals was obtained (Supplementary Table S2).

**Figure 3:**
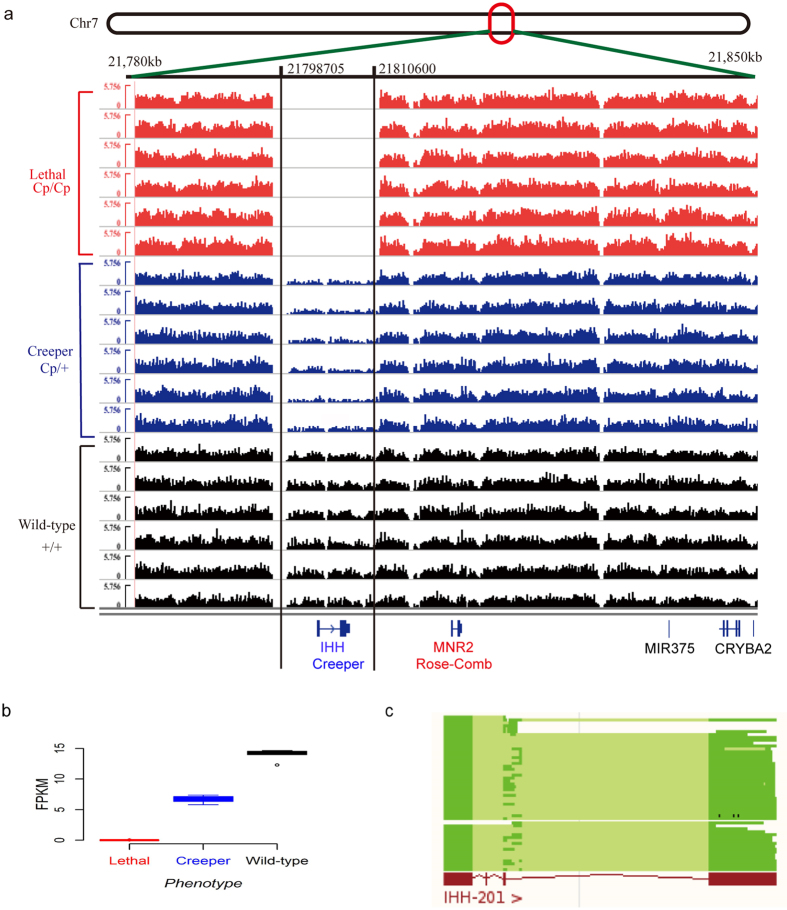
Identification of mutations underlying the semi-lethal Creeper trait by whole-genome sequencing. (**a**) Normalized genome coverage of the deletion region on chicken chromosome 7 for lethal embryos, Creeper and wild-type chickens. The deletion region (chr7: 21,798,705-21,810,600) includes the entire *IHH* gene. Full deletion of *IHH* was observed in the lethal embryos. Each group has 6 samples (N = 6). (**b**) FPKM (Fragments Per Kilobase Per Million Fragments Mapped on the chromosome, FPKM) was calculated for lethal embryos, Creeper and wild-type chickens. FPKM value follows the 0:1:2 ratio for lethal embryos, Creeper and wild-type chickens, respectively. (**c**) Comparison of *IHH* gene between vertebrates and chickens. Blue and red colors represent the cDNAs of *IHH* in vertebrates and chickens, respectively.

To identify the causative mutations responsible for the semi-lethal Creeper trait, we used a set of algorithms to detect various structural variations (SVs; including SNP, indel, rearrangement, translocation, inversion, tandem duplication, copy number variation) from the whole-genome sequencing data and directly probed the causative mutation from the SVs (Fig. 2). As the *Cp* and *Rose-comb* genes are closely linked^14^, we therefore prioritized the SVs on chicken chromosome 7 for investigation. We did not find any group-specific rearrangement, translocation, inversion, tandem duplication, medium-size indel or copy number variation (Supplementary Table S4–S6). There are six SNPs, five small indels and one large deletion which were solely presented in all Creeper individuals (Supplementary Table S3–S6). Among all the variations, only a 11,896 bp large deletion region (chr7: 21,798,705-21,810,600) covering the entire Indian hedgehog (*IHH*) gene (Fig. 3a) was top-ranked in terms of the genetic effects, chromosome position, and mutation type in our analysis pipeline (Fig. 2). Read depth was significantly lower in the deletion region than in both sides of the deletion region in the Creeper chickens, and the read depth in the deletion region of the Creeper chickens was almost half of that in the wild-type chickens (Fig. 3a). All 6 Creeper chickens were shown to carry the same *IHH* deletion. The *IHH* gene is adjacent to the *MNR2* gene which is a causative gene for *Rose-comb* mutation in the chicken^14^ (Fig. 3a). Our results strongly suggest that the deletion of *IHH* is the causative mutation for the semi-lethal Creeper trait.

### Deletion of *IHH* is completely associated with the semi-lethal Creeper trait

**Figure 4:**
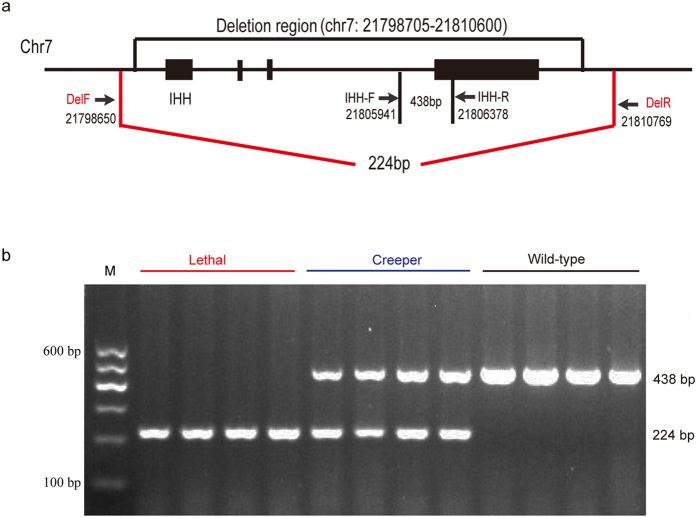
Schematic presentation of primer design and detection of *IHH* deletion by a diagnostic PCR test. (**a**) Diagnostic primers for wild-type and Creeper chickens. Expected size of PCR product of the wild-type chicken is 438 bp while for the Creeper chicken is 224 bp. (**b**) Diagnostic genotyping of three phenotypes. Lethal embryos has a single PCR product of 244 bp, Creeper chicken has two bands at 244 bp and 438 bp, and the wild-type chicken has a single band at 438 bp.

The breakpoint of the deletion region was confirmed by a diagnostic PCR test using a forward primer in the upstream and a reverse primer in the downstream of the deletion region (Fig. 4a). An expected, 224 bp PCR product was obtained from the lethal embryos (Fig. 4b and Supplementary Fig. S2). In comparison, an amplicon positioned within the deletion region yielded the expected 438 bp product in the wild-type chickens (Fig. 4b and Supplementary Fig. S2). PCR products from the heterozygotes had both 224 bp and 438 bp bands. PCR products of lethal and wild-type embryos were further confirmed by Sanger sequencing (Supplementary Fig. S2). It is clear that three different genotypic individuals can be clearly classified by the diagnostic PCR test for further analysis (Fig. 4b).

**Figure 5:**
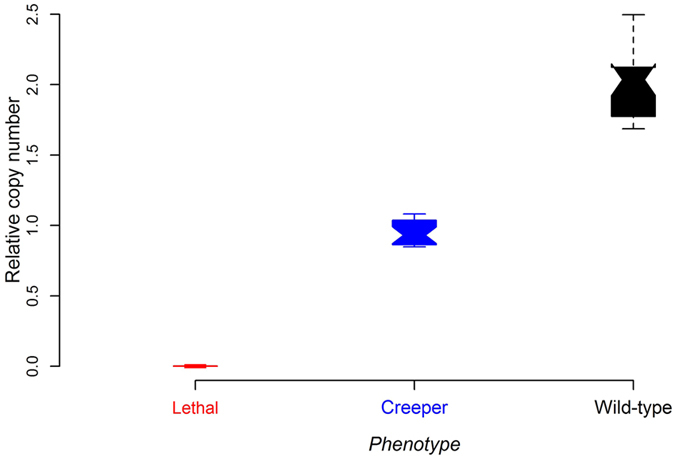
Relative copy number of *IHH* in genomic DNA from three different genotypic individuals by qPCR analysis. Red, blue, and black colors represent lethal embryos, Creeper and wild-type chickens, respectively. Data are represented as mean ± SD (N = 16).

Copy number of *IHH* gene in three genotypic individuals was examined by SYBR Green qPCR analysis with genomic DNA as the template. The results showed 0:1:2 ratio for lethal embryos (*Cp*/*Cp*), Creeper (*Cp*/+) and wild-type (+/+) chickens, respectively (Fig. 5).

To further explore the *IHH* deletion in a wide range of the Creeper populations by the diagnostic PCR test, a large segregated population from a mating of Creepers (*Cp/*+ × *Cp/*+) was constructed to test the association of genotypes with phenotypes. In total, 511 samples (embryos, n = 130; chickens, n = 381) of Creeper progeny were collected for association testing. Our result suggested that the complete association between the *IHH* deletion and the Creeper phenotype was observed in all the tested samples (*p* > 0.05, Table 2). The results of the complete association between deletion of *IHH* and the Creeper phenotype further suggest that this deletion is the causative mutation for the semi-lethal Creeper trait in chicken.

We genotyped all embryos found to be dead at E4 by the diagnostic PCR test and randomly chose 6 homogenous *Cp/Cp* samples for further whole genome sequencing (sequence coverage: 15–18X), as shown in Supplementary Table S2. No sequencing read was found in the deletion region (Fig. 3a), confirming the complete deletion of *IHH* in the early lethal embryos. The ratio of normalized read number of the three genotypes was close to 0:1:2 for lethal embryos, Creeper and wild-type phenotypes, respectively (Fig. 3b). Taken together, these lines of evidence allowed us to further conclude that a complete deletion of *IHH* causes a fully penetrant and dominant inheritance of the Creeper trait in chicken.

### Expression analysis reveals decreased *IHH* quantity responsible for the Creeper trait in chicken

**Figure 6:**
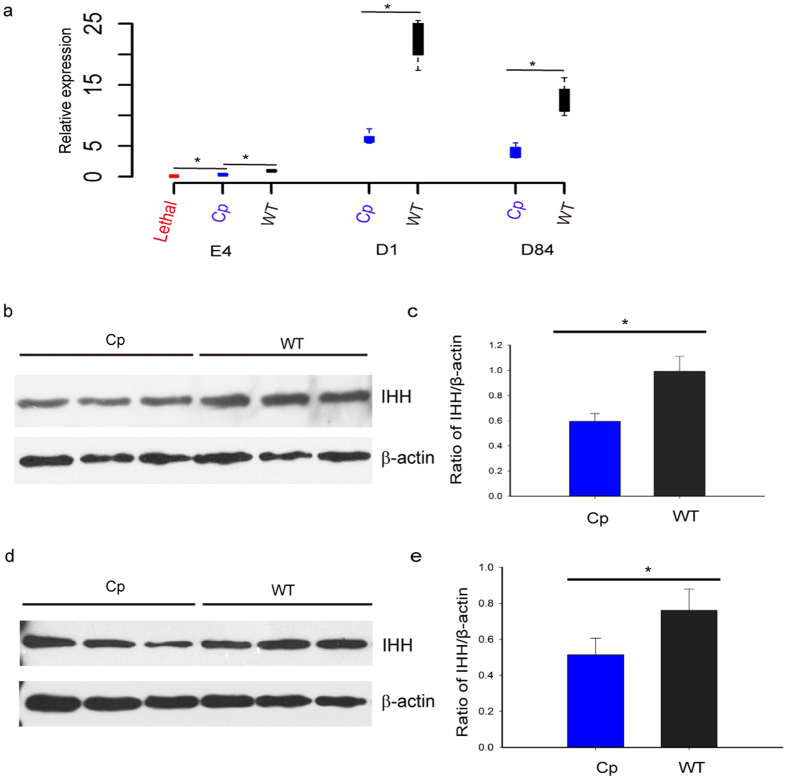
Expression analysis of *IHH* in tibial cartilages in three genotypic individuals. (**a**) qPCR analysis of *IHH* at E4 (embryonic day 4), D1 (day of hatch), and D84 (day 84 post-hatch) (N = 8 for each group). (**b,d**) Western blot analysis of IHH from Creeper and wild-type male chicks at D1 and D84. (**c,e**) The quantitative analysis of western blot results at D1 and D84 using Image J software. Western blot analysis showed the expression of IHH protein in chicken tibial cartilages. β-actin was used as a loading control. Three biological replicates were conducted in this experiment. Data are expressed as mean ± SD (N = 3). *p < 0.05.

To better understand the molecular mechanism underlying the semi-lethal Creeper trait in chicken, we investigated the expression pattern of *IHH* in early embryos and tibial cartilages from three genotypic individuals. E4 embryos were clearly divided into three genotypes using the diagnostic PCR test, then randomly chosen to perform expression analysis. Our qPCR analysis indicated that *IHH* was expressed at much lower levels at different embryonic developmental stages in the Creeper than in the wild-type chickens (Fig. 6a). Western blot analyses also detected lower IHH levels in tibial cartilages from the Creeper as compared to wild-type chickens (Fig. 6b–e). Our results showed that this deletion resulted in quantitative reduction of *IHH* expression, suggesting that decreased expression of *IHH* was incapable of providing sufficient protein product to maintain normal function and thus led to the Creeper trait in the heterozygotes while dominant homozygotes were lethal owing to loss of the whole gene product.

## Discussion

The Creeper trait is a well-known monogenic phenotype in chicken following Mendelian autosomal dominant inheritance^7^. However, the molecular basis of this trait remains poorly understood. It will be of great importance to identify genes and/or causative mutations affecting the Creeper trait, understand the biological and medical significance of the genes, and determine the gene regulatory mechanisms underlying this trait. In the present study, we demonstrate that deletion of *IHH* is responsible for the Creeper trait and associated early embryo death in chicken.

By extensive bioinformatics analyses, we identify that a large deletion region ranging from 21,798,705 to 21,810,600 on GGA7 harbors the entire *IHH* gene in the Creeper chickens. IHH is a member of the hedgehog family, which is a conserved signaling family in vertebrates and some invertebrates^18^ (Fig. 3c). In higher vertebrates, there are at least three highly similar hedgehog genes including Sonic hedgehog (*SHH*), Desert hedgehog (*DHH*), and *IHH*^19^,^20^. *IHH* is mainly expressed in the developing cartilage elements, indicating that it plays pivotal roles in regulating numerous developmental processes of bone formation^21^. It has been suggested that *IHH* is essential for endochondral bone formation and coordinates the proliferation and differentiation of chondrocytes, and osteoblast differentiation^22^,^23^,^24^,^25^. Single-point mutations in the *IHH* gene can cause the brachydactyly type A-1(BDA-1) with shortening or missing of middle phalanges^26^,^27^ and the severe skeletal dysplasia named acrocapitofemoral dysplasia (ACFD) in humans^28^. Genetic studies also demonstrated that deletion of one amino acid in IHH resulted in mild BDA-1 in a small Dutch family^29^. In the mouse model, most knock-out mouse embryos (*IHH*^^−^/^−^^) die before birth while the heterozygotes (*IHH*^+/−^) survive but exhibit foreshortened forelimbs and unsegmented or uncalcified digits after birth^30^, which is highly similar to what we observed in the Creeper chickens. As one of the key genes driving animal body development, *IHH* is conserved in gene function and signaling pathway in the major animal clades^25^ and is required for embryonic bone formation in development^31^. Moreover, we also found that the deletion of *IHH* is closely associated with the Creeper trait in chicken following the complete Mendelian segregation. These pieces of evidence strongly suggest that *IHH* is a causative gene responsible for the Creeper trait.

Although *IHH* plays significant roles in bone formation, skeletal morphogenesis, and gut development, and mutations in *IHH* cause abnormal digital development and morphogenesis problem in human and mouse^26^,^27^,^28^,^30^, little is known about the effect of *IHH* deletion in chicken cartilages. In the present study, we demonstrated that decreased expression of *IHH* in the Creeper chicken cartilages affected bone development. Effects of allele deletion in the heterozygous progeny cannot be masked by one wild-type allele in this case. It has been widely accepted that haploinsufficiency is the genetic mechanism for loss-of-function mutations in most autosomal dominant disorders, which is discovered in all eukaryotes from yeast to humans^32^. Genetically, haploinsufficiency refers to a dominant phenotype in diploid organisms which are heterozygous for the deletion of one functional gene copy, leading to an abnormal phenotype or disease states^33^. In humans, several reported disorders are caused by haploinsufficiency mutations, such as autoimmune lymphoproliferative syndrome^34^, immune dysregulation^35^, cognitive abnormality^36^, and even enhanced cancer susceptibility^37^,^38^. Previous studies have also shown the effect of haploinsufficiency mutations for abnormal phenotypes in model organisms. In Drosophila, the *Minute* mutations causing numerous developmental abnormalities are good examples to interpret the effects of ribosomal haploinsufficiency in multicellular eukaryotes^39^,^40^. A study on zebrafish demonstrated that 11 ribosomal genes haploinsufficiency mutations resulted in increased susceptibility to tumor formation^41^. Therefore we assume that *IHH* haploinsufficiency is insufficient to provide enough gene product to sustain normal function, thus resulted in the Creeper trait in the heterozygotes while dominant homogenous embryos were lethal during the early stage of embryonic development in chicken. Further experimental studies on heterozygotes are necessary to determine that the Creeper trait is the haploinsufficiency disorder in large populations.

Autosomal dominant disorders are detrimental and rare in domestic animals. Examples of such diseases are wattles in swine^42^; epidermolysis bullosa in Danish Hereford calves^43^; congenital myotonia in goat^44^; hyperkalemic periodic paralysis in horse^45^; autosomal dominant progressive retinal atrophies in Bullmastiff and English mastiff dogs^46^,^47^; and collagen dysplasia in cat^48^. Here we add our Creeper phenotype to this list and provide another important case for illustrating autosomal dominant inheritance in animal genetics. It is considered that chickens have been used as a good and traditional model for studying embryonic vertebrate development as their embryos can be easily manipulated *in vitro*^49^. Using the Creeper chicken as a model, the expression of *IHH* and the status of bone formation can be dynamically monitored. The Creeper chicken thus provides another significant feature to serve as an attractive and unique model for studying *IHH* function and haploinsufficiency disorders.

To the best of our knowledge, this is the first report answering the longstanding riddle about the Creeper trait in animal genetics. Our data strongly suggest that the deletion of *IHH* is the causative mutation for the Creeper trait and associated early embryo death in chicken. This study will not only highlight the biological role of *IHH* in animal development, but also shed light on why chicken provides a valuable and unique model to examine the genetic basis and biological processes that likely underpin phenotypic mutations in humans and other species.

## Materials and Methods

### Ethics statement

All animal experiments were reviewed and approved by the Institutional Animal Care and Use Committee of China Agricultural University (permit number: SYXK 2007–0023). All experimental protocols and procedures were carried out according to relevant regulations and guidelines established by this committee, and all efforts were made to minimize the suffering of the chickens.

### Chicken population and sample preparation

Chinese Xingyi bantams were obtained from the Guizhou Xingyi Bantam Conservation Farm and reared in the Poultry Genetic Resources and Breeding Station at China Agricultural University. Each chicken was wing-banded. Adult chickens were reared individually in single cages. All chicken photographs (Figs 1a–e and 2) were obtained from the station. Radiographic analysis of photographs (Fig. 1c–e) was performed using DicomPACS Digital X-ray System and Imaging Solutions (SEDECAL, Madrid, Spain).

We constructed a cross between Creeper chickens to generate different phenotypic progenies for association testing, incubation experiments, and tissue sampling (Supplementary Table S1). We conducted two trials for phenotyping and DNA/RNA sample collection. All chicks were pedigreed according to intercross information. In the incubation experiment (first trial), we incubated fertile eggs from the Creeper population and a control cross between Creeper and wild-type birds (Table 1 and Supplementary Table S1). In the second trial, we collected all 511 fertilized eggs with viable embryos from the Creeper cross for association study (Table 2). DNA was extracted from embryos at different incubation periods. We did three manual candlings to identify early death embryos at E4, mid-term death at E10 and late death at E19. We measured shank length using a digital vernier caliper at every two weeks from hatch to 20 weeks of age. Creeper chickens can be clearly classified by shank length at day of hatch. We chose chicks from full-sibs with different phenotypes (Creeper and wild-type chickens). Twelve birds derived from a mating of Creeper chickens, including 6 Creeper chickens (*Cp*/+) and 6 wild-type chickens (+/+), were chosen for whole-genome sequencing, as phenotyped by shank length at 4 weeks of age (Creeper chicken: <4.08 cm (mean-SD); wild-type chicken: >5.18 cm (mean + SD)). This design decreased the noise of genetic background, which may complicate the following analyses. In total, 3 pairs of full-sib females and 3 pairs of full-sib males from 4 sire families were chosen for whole-genome sequencing.

Blood samples were collected from the wing vein and stored in acid citrate dextrose (ACD) anticoagulant at −20 °C prior to DNA extraction. Genomic DNA from whole blood was extracted by standard phenol-chloroform methods. Embryonic DNA was extracted from whole embryos using PureLink® Genomic DNA Kits (Invitrogen).

### Whole-genome library construction and sequencing

Paired-end libraries with average insert size of approximately 500 bp were constructed for each sample according to the manufacturer’s instructions (Illumina, San Diego, CA). Library quality and concentration were determined using an Agilent 2100 Bioanalyzer (Agilent Technologies, Palo Alto, CA) and Qubit 3.0 Fluorometer (Life Technologies, CA, USA). These libraries were subjected to 2 × 100 bp paired-end (PE100) sequencing on a HiSeq2000 instrument (Illumina). A standard Illumina base-calling pipeline was used to process the raw fluorescent images and the called sequences. Read quality was evaluated using the FastQC package (www.bioinformatics.babraham.ac.uk/projects/fastqc/). For genome re-sequencing data, short-reads were trimmed 15 bp from the 3′-end according to the base quality distributions. The raw sequencing data reported in this paper have been publicly deposited in the NCBI Short Reads Archive (SRA) with accession number SRP047477.

### Whole-genome sequencing data analysis

Paired-end short reads were aligned to the *Gallus gallus* reference genome (*Galgal* 4) using the Burrows-Wheeler Aligner (BWA, version 0.6.2) algorithm with default parameters^50^. SAMTools (version 0.1.19)^51^ was used to remove duplicate reads that might have been caused by PCR. To improve the accuracy of reads alignment, aligned reads were realigned at putative SNPs and indel positions using the Genome Analysis Toolkit (GATK, version 2.5.2) realigner algorithm^52^. Base quality scores were recalibrated using the GATK recalibration algorithm. The options used for SNP and indel calling were a minimum 5-read mapping depth and mapping quality of 20. As indels ranged from 1 to 1000 bp, we used three different algorithms to search for indels. The GATK software is suitable for detecting small indels. Mate-Clever is more suitable to probe the medium-size indels from the resequencing data than GATK^53^ and was thus used to find the medium-size indels. We used Pindel (version 0.2.4t)^54^ to detect large structural variations (SVs). Copy number variation (CNV) was called using the CNVnator (version 0.2.7)^55^. Window bin-size was set to 100 bp with the GC-content adjustment, and the mean-shift algorithm was used to infer the CNV with the *p* < 0.01. Once we obtained all the SVs from the different algorithms/software, we used the VCFTools (version 0.12b)^56^ to extract the common variations for Creeper or wild-type group. SV was filtered by 20 reads for large indels and 8 reads for small SVs. The final SV supported in all 6 individuals of each group was considered to be the potential causative mutations for the Creeper trait. Genetic effects of the SV was assessed by their variation type and marked as HIGH, MODERATE and LOW effects for each SV suggested by SnpEff (version 3.4)^57^. In general, large chromosomal deletions, exon deletions, frame shifts and lost/gained stops were thought to have strong genetic effects on phenotypes. Among these variations with potentially strong genetic effects, large deletion/insertion was ranked on the top. In addition, SV on chromosome 7 was set to first-class for further genetic analysis, as previous studies showed that structural rearrangement of *MNR2* gene causing Rose-comb in chicken is closely linked with the semi-lethal Creeper trait (Fig. 3a).

### Diagnostic genotyping and Sanger sequencing validation

The delF and delR primers located upstream and downstream of the deleted region were utilized to amplify 224 bp fragment, while *IHH*-F and *IHH*-R primers annealing to *IHH* were used to produce 438 bp fragment. Two pairs of primers were designed to amplify a series of specific bands (Supplementary Table S7). The PCR reactions were carried out in a reaction volume of 20.0 μl containing 100 ng genomic DNA, 2.0 μl of 10 × *Taq* polymerase buffer, 400 μM dNTPs, 1.5 U *Taq* DNA polymerase (Tiangen Biotech, Beijing, China), 2.0 μM of each delF and delR, and 4.0 μM of each *IHH*-F and *IHH*-R primer. The diagnostic PCR protocols included 94 °C for 5 min, 35 cycles of 94 °C for 30 s, 57 °C for 30 s, 72 °C for 35 s, and a final extension at 72 °C for 10 min. The PCR products were separated by 2.0% agarose gel electrophoresis. Sanger sequencing validation was performed for two different PCR products, which were analyzed using BLAT (http://genome.ucsc.edu/cgi-bin/hgBlat) tools to cross-validate the expected sequences and reference sequences.

### Quantitative PCR confirmation of *IHH* deletion

A total of 48 DNA samples, including 16 lethal embryos, 16 Creeper and 16 wild-type birds, were examined using qPCR to quantify DNA in the three different genotypes. All lethal embryos were further validated to be homozygotes of *Cp/Cp* by the diagnostic PCR test. PCR primers were designed using the Primer3web software (http://primer3.ut.ee/) and UCSC In-Silico PCR to examine the specificity and sensitivity (http://genome.ucsc.edu/). The single-copy *PCCA* gene, previously validated as a non-CNV locus, was selected as an internal control^58^. The relative copy number was calculated using the 2^−ΔCt^ method.

### mRNA expression analysis by qPCR

Tibial cartilages were taken from heterozygous (*Cp*/+) and wild-type (+/+) male birds at day of hatch (D1, N = 8) and day 84 post-hatch (D84, N = 8). Early lethal embryos were collected and validated by genotyping at the fourth day of incubation (E4, N = 8). All the tissue samples and embryos were stored in RNAlater (Ambion, Austin, USA). Total RNA was extracted using RNA Mini kit (Life Technologies, Carlsbad, USA) and purified by RNeasy Mini Kit (Qiagen, Hilden, Germany). Briefly, 1 μg total RNA was used for first strand cDNA synthesis using TransScript gDNA Remover RT kit (TransGen, Beijing, China) according to the manufacturer’s protocols. All primers spanning at least one intron were designed for qPCR by Primer Premier 5.0 software (Premier Biosoft, Canada). The primer sequences are listed in Supplementary Table S7. All samples were run in triplicate using cDNA for qPCR using an ABI Prism 7500 instrument (Applied Biosystems, Carlsbad, CA). The expression data were normalized using *GAPDH* as an endogenous reference gene and calculated using the 2^−ΔΔCt^ method.

### Western blot

Tibial cartilages were obtained from Creeper and wild-type male chickens at D1 and D84 (N = 3 for each group at each developmental stage), respectively. Total proteins were extracted using RIPA buffer (Beyotime, Nanjing, China) following the manufacturer’s protocols. Total protein concentrations were measured using the BCA protein kit assay (Sigma-Aldrich, St. Louis, USA). Protein samples were separated by 12.0% SDS-PAGE and electro-transferred to PVDF membrane (Millipore, Billerica, USA). The membranes were blocked for 1 h at room temperature and incubated overnight at 4 °C with rabbit anti-IHH (1:1000, Novus Biologicals, Littleton, USA) and rabbit anti-β-actin (1:1000, Cell Signaling Technology, Beverly, USA), followed by HRP-conjugated anti-rabbit IgG (1:1000, Novus Biologicals, Littleton, USA) for 1 h. The enhanced chemiluminescence detection kit (Beyotime, Nanjing, China) was used to visualize the immunoreactive proteins. Quantification and data analysis were conducted using Image J software^59^.

### Statistical analyses

Statistical analyses were conducted under the R computation environment (www.r-project.org). The two-tailed student’s *t-*test was used to compare the mRNA expression level and DNA quantification between samples. Segregation ratios obtained from different matings were analyzed by *Chi*-square tests. Data were expressed as mean ± SD. Differences were considered to be statistically significant at *p* value < 0.05.

## Additional Information

**How to cite this article**: Jin, S. *et al*. Deletion of Indian hedgehog gene causes dominant semi-lethal Creeper trait in chicken. *Sci. Rep.* **6**, 30172; doi: 10.1038/srep30172 (2016).

## Supplementary Material

Supplementary Information

## Figures and Tables

**Table 1:** Summary of embryonic mortality during the entire period of incubation.

Mating	Fertile eggs^1^	Early embryonic death^2^ (N)	Total embryonic death^3^ (N)	Early mortality (%)	Total mortality (%)
Cp/+ (♂) × Cp/+ (♀)	410	120	142	29.27	34.63
Cp/+ (♂) × +/+ (♀)	304	14	32	4.61	10.53

^1^Fertile eggs were determined using candling method.

^2^Early embryonic death was measured by candling at E4.

^3^Total embryonic death was determined by counting all dead embryos during the incubation period.

**Table 2:** Diagnostic genotyping of individuals from the Creeper intercross population.

Description	Genotype		
	*Cp/Cp*	*Cp/*+	+*/*+
Observation (N^1^)	130	258	123
Expectation (N)	131.3	255.5	124.3
*Chi*-square test	*χ*^2^ = 0.0509, *p* = 0.8215		

^1^N: the number of embryos and chickens.
