# Supplementary material for: Deletion of Indian hedgehog gene causes dominant semi-lethal Creeper trait in chicken
Source: Sci Rep. 2016 Jul 21;6:30172. doi: 10.1038/srep30172 (PMC4954956; doi:10.1038/srep30172)
Supplement: Supplementary Information [file srep30172-s1.pdf]

Supplementary information

Deletion of Indian hedgehog gene causes dominant semi-lethal Creeper trait in chicken

Sihua Jin[1], Feng Zhu[1], Yanyun Wang[1], Guoqiang Yi[1], Junying Li[1], Ling Lian[1], Jiangxia Zheng[1], Guiyun Xu[1], Rengang Jiao[2], Yu Gong[3], Zhuocheng Hou[1,*], Ning Yang[1,*]

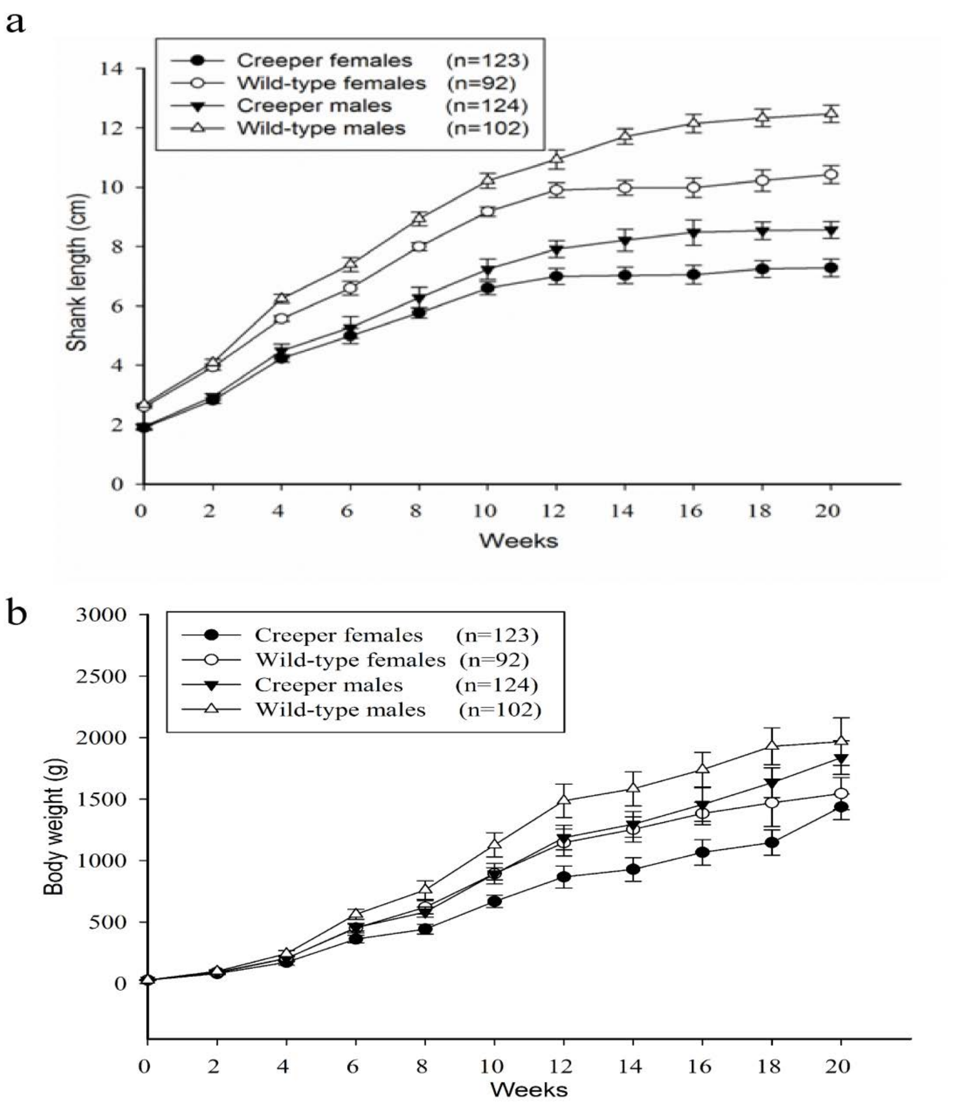

Figure S1. Dynamic changes in shank length and body weight of Creeper and wild-type fowls from 0 to 20 weeks of age. (a) The shank length of wild-type fowls is significantly longer than that of Creeper birds from 0 to 20 weeks of age. (b) Wild-type chickens are significantly heavier than Creeper birds from 2 to 20 weeks of age. Data were analyzed for males and females separately in each week. Data are presented as mean ± SD (standard deviation).

a

AGCCCCTCATTGTTGTCTCAGCGAGTTAATCAGATGCCATAATCCTGTTGTGGAAATGGGGGCAGTCCC
TCACCGGCGGGCACAGGCGCAGAGTGGTCTGTGCTGAGCCAACACTCCAGGAATTTAATTTGTTTAAT
ATATTCCCTGGGAAAGAAGAAAATAAATAAGTGGCACCTTGCGTGCCCCCATGGCTCACTCTCGCAACG
GAGGTGTCAGCTTAACGA

b

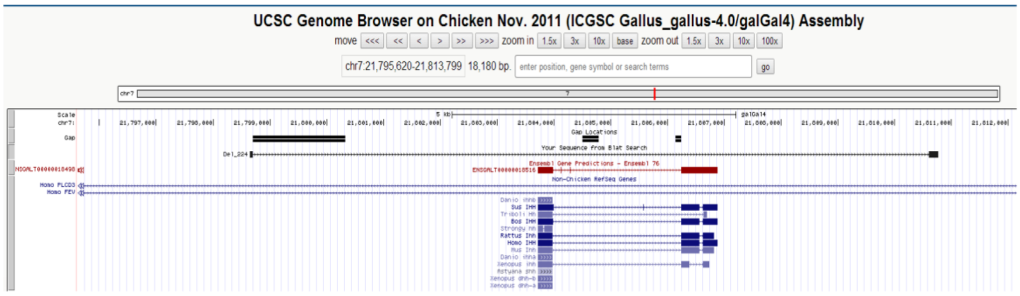

c

CTGCCTTGTGCGTTCTCACTGCCCTTTGCCCTGGGGCTGCCCTTTGCCCTGGGCGCTCCCTGTGCCAGA
AGGTTTGGGGACCCTGCTTTGCAGAGCAGCACCGCGGTGGGTCACGGGCACTGGCAGGCACAGCTCGGC
ACCCCTGGCGGCACGGTGGCAGACGCGGGGCTGCGTGCAGGGAGCTGTGCGCATCGGCAATGCCAGGGC
AGTGTTATCCTTGCCGGGCAGCACGGGGCCTCCGTGCGTCGTGCAGAACCCTCTCCTTTGCTCTCTCCCCGT
GCAGAGCACTCGGCTGCCGCGAAGACGGGCGGCTGCTTCTCCGGGCGGGCGCTGGCGACGCTGGAGAAC
GGTGCCCGGACGCCACTGTGGGCACTGCGGCCGGGCCAGCGGGTGCTGGCGATGGACGGGGCGGGCCG
GCC***CACCTACAGCGACTTCCTG***

d

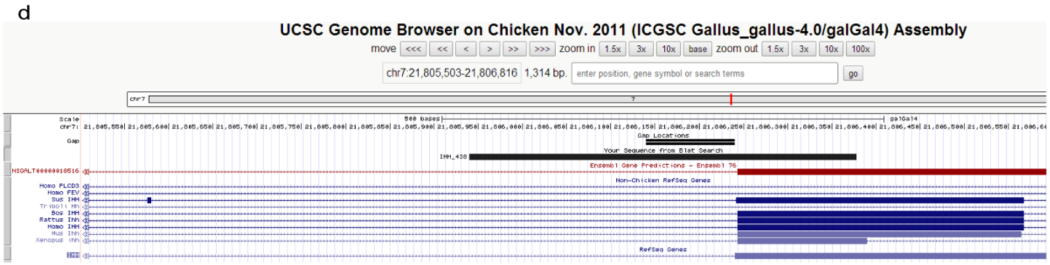

Figure S2. PCR products of primers for the deletion region and BLAT results for Creeper and wild-type chicken groups. (a) PCR products from the lethal embryos. (b) BLAT results of the PCR products. Results showed that PCR products covered the entire *IHH* region. (c) Red color represents the primers. All fragments show PCR products from the *IHH* region. Yellow base represents the “N” sequences in the reference genome, which were successfully sequenced in this study. (d) BLAT results of the PCR products. Results of the PCR products are the same as expected size and position.

Table S1. Segregation of the Creeper trait and hatchability obtained from two mating of Creeper *inter se* and of Creeper with wild-type chickens

Mating	Parents		Year of mating	Phenotype			Hatchability
	Male (♂)	Female (♀)		Lethal	Creeper	Wild-type	(%)
Creeper (♂) × Creeper (♀)	3	15	2012	35	45	22	65.7
	9	45	2013	150	176	85	63.5
	6	30	2014	135	185	86	66.7
			Total	320	406	193	65.2
			Expectation		399.3	199.7	
			Chi-square test		χ^2=0.1231, p=0.7527		
Creeper (♂) × Wild-type (♀)	3	15	2012	10	43	30	88.0
	7	35	2013	39	150	152	88.6
	6	30	2014	29	145	125	90.3
			Total	78	338	307	89.2
			Expectation		322.5	322.5	
			Chi-square test		χ^2=0.6523, p=0.4193		

Table S2. Basic statistics of the short-reads quality and mapping.

Phenotype	Sample ID	Total reads	Mapped reads	Properly paired reads	Mapping rate (%)	Coverage(%)	Q20(%)	Reads depth (X)
Lethal	5661	194,404,600	189,151,232	184,425,580	97.30	97.87	95.91	18.13
	5666	180,633,586	175,799,388	172,032,464	97.32	97.91	96.10	16.84
	5668	187,984,548	182,764,274	178,528,940	94.97	97.91	96.30	17.53
	5665	158,812,964	154,378,202	150,380,644	94.69	97.80	96.41	14.81
	5657-1	165,703,848	161,350,285	157,892,858	97.37	97.35	95.65	15.45
	5657-2	182,726,008	177,444,950	173,281,198	94.83	97.92	96.07	17.04
Creeper	61367	176,853,104	173,306,711	170,508,974	97.99	97.39	96.51	16.49
	61375	162,325,872	158,730,001	155,931,334	97.78	97.77	96.05	15.13
	61446	148,441,490	145,024,009	142,085,118	97.70	97.29	96.30	13.84
	61455	147,578,036	144,277,570	141,461,982	97.76	97.73	96.49	13.76
	61460	158,174,222	154,797,665	151,994,542	97.87	97.34	96.35	14.75
	61468	161,436,618	157,625,919	154,418,498	97.64	97.85	96.42	15.05
Wild-type	61369	155,448,076	152,359,501	150,007,122	98.01	97.32	96.35	14.49
	61376	178,089,842	174,142,402	170,873,432	97.78	97.82	95.80	16.60
	61450	126,763,899	126,349,870	121,656,192	97.95	97.19	96.21	11.54
	61458	171,273,608	167,485,263	164,577,282	97.76	97.88	96.61	15.97
	61462	166,591,854	163,102,595	160,383,564	97.91	97.38	96.76	15.53
	61466	168,086,292	164,150,199	160,929,614	97.66	97.84	96.52	15.67

Total reads were the reads number after quality filtering according to raw data analysis pipeline. Mapped reads was the total reads number which could be mapped onto the reference genome. Properly paired reads was counted by SAMTools which counted paired reads information and inferred the properly paired based on the average insert size. Mapping rate (%) is the ratio of mapped reads number to sequence reads number. Coverage was calculated as the percentage of mapped reference genome with respect to the entire genome. Q20 (%) is the ratio of quality base-pairs higher than 20 over sequenced base-pairs. Reads depth, the ratio of the number of base that had been sequenced relative to the total bases in the entire genome.

Table S3. SNPs with moderate and high potential genetic effects in the Creeper group.

Chr.	Position	Ref[1]	Alt.	Freq.	RD	Type for CDS	Transcript	Effect
6	19011030	G	A	1	105	splice_acceptor_variant	ENSGALT00000010448	HIGH
6	14136414	T	C	1	69	missense_variant	ENSGALT00000007999	MODERATE
12	9286456	C	G	1	65	missense_variant	ENSGALT00000035408	MODERATE
22	2256548	T	G	1	47	missense_variant	ENSGALT00000005118	MODERATE
22	2256549	T	C	1	46	missense_variant	ENSGALT00000005118	MODERATE
22	2256550	T	G	1	45	missense_variant	ENSGALT00000005118	MODERATE

[1]Ref: the nucleotide in the reference genome; Alt: the nucleotide in the Creeper birds. Freq. is the genotype frequency in the Creeper birds. We kept only those variations fixed in the Creeper group. RD: reads depth. Type: classified by the SnpEff software. Effect: potential genetic effects to the phenotype which is classified by the SnpEff software. In general, the large deletion on chromosome 7 and exon deletion were ranked as the HIGH genetic effect for the phenotype.

Table S4. Inferred small indels by GATK software in the Creeper group.

Chr.	Position	Ref.	Alt	Freq.	RD	Type for CDS	Transcripts	Effect
1	14621569	GCCGCCCTTCCCTT	G	1	22	frameshift_variant	ENSGALT00000043578	HIGH
3	12844310	GTTCCT	G	1	20	frameshift_variant	ENSGALT00000042971	HIGH
3	42745189	TC	T	1	33	frameshift_variant	ENSGALT00000045188	HIGH
1	59454525	A	ATGAGTCTCTTTTTGAGCT	1	83	disruptive_inframe_insertion	ENSGALT00000021101	MODERATE
27	1547460	A	AAGGGCG	1	47	inframe_insertion	ENSGALT00000040725	MODERATE

Chr.: chromosome; Ref: the nucleotide in the reference genome; Alt: the nucleotide in the Creeper group. Freq.: the genotype frequency in the Creeper birds. We kept only those variations fixed in the Creeper fowls. RD: reads depth. Type for CDS: genetic effects on the coding sequence region and classified by the SnpEff software. Effect: potential genetic effects for the phenotype which is classified by the SnpEff software. In general, the large deletion on chromosome and exon deletion were ranked as the HIGH genetic effect for the phenotypes.

Table S5. Statistics of the medium-size indels and CNV.

Group	Sample ID	Indels	CNV		Group-specific Indels	Group-specific CNV
			Duplication	Deletion		
Creeper	61367	88,568	218	590	0	0
	61375	67,452	235	568		
	61446	147,685	202	486		
	61455	83,559	229	492		
	61460	77,421	204	555		
	61468	96,568	224	572		
Wild-type	61369	71,973	236	511	0	0
	61376	100,200	214	627		
	61450	90,269	215	496		
	61458	102,267	230	512		
	61462	137,178	213	478		
	61466	94,480	233	493		

Mate-Clever is good for probing medium-size indels (30-200 bp). No commonly medium-size indels were observed within each group. CNV: copy number variation. CNV was called by CNVnator.

Table S6. Structural variations inferred by Pindel in the Creeper group.

Chr.	Start	End	Ref length	Alt. length	SV Type	RD	Type for CDS	Transcripts	Effect
7	21798705	21810600	0	-11896	RPL	23	exon_loss_variant, stop_lost	ENSGALT00000018516	HIGH
5	49480890	49480890	2	1	INS	26	frameshift_variant	ENSGALT00000018544	HIGH

Chr.: chromosome. Ref length: the nucleotide length in the reference genome; Alt length: the nucleotide length in the Creeper group. RD: reads depth. SV Type: structural variation defined by Pindel. RPL, deletion; INS, insertion. We kept only those variations fixed in the Creeper flock. Type for CDS: genetic effects on the coding sequence region and classified by the SnpEff software. Effect: potential genetic effects for the phenotype which is classified by the SnpEff software. In general, the large deletion on chromosome 7 and exon deletion were ranked as the HIGH genetic effect for the phenotype.

Table S7. Primer sequences used in this study.

a. Primers used for diagnostic genotyping by a diagnostic PCR test

Primer name	Sequences (5' to 3')	Length (bp)
IHH	*IHH*-F: CTGCCTTGTGCGTTCTCA	438
	IHH-R: CAGGAAGTCGCTGTAGGTG	
delF/R	delF: AGCCCCTCATTGTTGTCTCA	224
	delR: TCGTTAAGCTGACACCTCCG	

b. Primers used in qPCR analysis for DNA samples

Gene	Sequences (5' to 3')	Length (bp)
IHH	F1: GGGAGGGCATCGCATAGAA	113
	R1: GGAACACCGTCTGACCCAGTTA	
PCCA	F: CAGACACACAGAGCCCATCTCT	65
	R: TGGAGCAGTGGTGGCTGTT	

c. Primer sequences used for quantitative PCR

Gene	Sequences (5' to 3')	Length (bp)
IHH	F2: CGCTTTGTGGGGTGATGC	111
	R2: TCCGTACAAGGCTCTGGTTT	
GAPDH	F: CGTCCTCTCTGGCAAAGTCC	132
	R: TTCCCGTTCTCAGCCTTGAC	
